# Rs4846049 Polymorphism at the 3′-UTR of MTHFR Gene: Association with Susceptibility to Childhood Acute Lymphoblastic Leukemia

**DOI:** 10.1155/2019/4631091

**Published:** 2019-10-13

**Authors:** Xiaolei Li, Shunguo Zhang, Feng Yu

**Affiliations:** ^1^School of Basic Medicine and Clinical Pharmacy, China Pharmaceutical University, Nanjing, Jiangsu Province 211198, China; ^2^Shanghai Children's Medical Center Affiliated to Shanghai Jiaotong University School of Medicine, Shanghai 200127, China

## Abstract

**Background:**

Accumulating evidence has suggested the polymorphisms of methylenetetrahydrofolate reductase (MTHFR) were associated with susceptibility to childhood acute lymphoblastic leukemia (ALL). However, the known conclusions of currently known polymorphic loci (677 C > T and 1298 A > C) remain controversial. This study was to investigate new genetic biomarkers for ALL by analyzing the MTHFR polymorphisms at the 3′-untranslated region, which is a location bound by miRNAs.

**Methods:**

Polymorphisms of rs4846049 (miR-555 binding) were assessed by PCR amplification and direct sequencing in 110 ALL patients and 105 healthy controls. The relative expression of MTHFR was detected by qRT-PCR.

**Results:**

Overall, genotype distribution or allele carrier frequencies were not significantly different between patients with ALL and healthy controls (*P* > 0.05). Subgroup analysis results showed that T allele (OR = 0.134, 95% CI = 0.028–0.639; *P*=0.005) or genotypes with T allele (TT + GT) (OR = 0.133, 95% CI: 0.024–0.727; *P*=0.017) may be a protective factor for ALL susceptibility in patients with age >8 years. This conclusion was also true for the group only focusing on the precursor B-cell ALL patients. Furthermore, karyotype abnormality was more commonly observed in patients with the GG genotype (56.0%) compared to carriers of TT (0%) or GT (40.6%) genotypes, while c-myc break frequency was significantly higher in TT carriers (33%) than that of patients with GT (3.1%) or GG (0%) genotypes. PCR analysis showed patients carrying the GG genotype of rs4846049 exhibited the reduced mRNA expression of MTHFR.

**Conclusion:**

MTHFR rs4846049 polymorphism may be associated with increased risk of childhood with ALL and MTHFR mRNA expression.

## 1. Introduction

Acute lymphoblastic leukemia (ALL) is the most common hematological malignancy in childhood, with an estimated incidence of approximately 2 cases per 100,000 person-years [1]. ALL is characterized by rapid proliferation and subsequent accumulation of immature T or B lymphoid precursors in the bone marrow, which contributes to a reduction in hematopoiesis and then causes infection, hemorrhage, and death of childhood [2, 3]. Thus, it is urgently needed to understand the pathogenesis of ALL for timely screening the high-risk patients in order to prevent poor prognosis.

Although the etiology of ALL remains not well understood, increasing evidence has suggested genetic variation in the methylenetetrahydrofolate reductase (MTHFR) gene plays critical roles [4, 5]. MTHFR encodes an enzyme that catalyzes the irreversible conversion of 5, 10-methylenetetrahydrofolate (5, 10-MTHF) to 5-methyltetrahydrofolate (5-MTHF), the main circulatory form of folate [6]. 5-MTHF causes the remethylation of homocysteine to methionine, a precursor of S-adenosylmethionine (SAM) that provides the methyl group for methylation of DNA [6]. DNA hypomethylation is an important contributor for activation of proto-oncogenes and induction of genomic instability, ultimately initiating carcinogenesis [7, 8]. Therefore, genetic variants that result in reduced expression or enzyme activity of MTHFR may be underlying mechanisms for the development of ALL.

Recently, two nonsynonymous single nucleotide polymorphisms (SNPs) in the MTHFR gene have been revealed to be associated with decreased enzyme activity, including C677T (rs1801133, alanine-to-valine substitution at codon 222 within the *N*-terminal catalytic domain of exon 4) and A1298C (rs1801131, glutamate-alanine substitution at codon 429 within SAM regulatory domain of exon 7) [9, 10]. Therefore, 677T (or CT, TT genotype) or 1298C (or CC, CA genotype) allele may be a risk factor for the development of ALL, which has been demonstrated in several studies [11–13]. However, conflicting conclusions were also reported by other scholars. Some studies suggested 677T or 1298C allele may be as a protective factor [4, 14], while the other indicated no association of these two SNPs with childhood ALL [14, 15]. Accordingly, there is still a need to investigate some new genetic biomarkers for ALL.

MicroRNAs (miRNAs) are small (20–25 nucleotides), endogenous noncoding RNAs that mediate mRNA degradation and translation repression through binding to the complementary sites on the 3′-UTR of target mRNAs. Theoretically, genetic variations in the 3′-UTR may alter the expression of MTHFR and also affect the susceptibility to diseases, which has been confirmed in other cancers [16–18], but not in ALL. This present study was, for the first time, to investigate the correlations between functional variants of the MTHFR gene at 3′-UTR and the risk of childhood ALL using a case-control study in a Chinese population.

## 2. Methods

### 2.1. Patients

A total of 215 subjects including 110 pediatric ALL and 105 healthy controls who received routine medical examinations in the same period were enrolled from December 2017 to September 2018. Moreover, demographic and clinical data, including age, sex, count of white blood cell (WBC), hemoglobin (Hb), and platelet at diagnosis, French-American-British (FAB) classification, the status of organomegaly, lymphadenopathy, cerebrospinal fluid (CSF) involvement, immunophenotype, karyotype aberrations (including chromosome number and structure changes), fluorescence in situ hybridization (FISH) results, and 2016 World Health Organization (WHO) classification [19] according cytogenetic and FISH analyses were collected from the clinical medical records.

All procedures performed in studies involving human participants were in accordance with the Institutional Review Board of Shanghai Children's Medical Center Affiliated to Shanghai Jiaotong University School of Medicine. The entire research was in compliance with the terms of the 1964 Helsinki Declaration and its later amendments or comparable ethical standards. Informed consent was obtained from the parents of all individual participants included in the study.

### 2.2. Polymorphism Selection

Online bioinformatics software (http://www.bioguo.org/miRNASNP/ and http://bioinfo.life.hust.edu.cn/miRNASNP2/) was used to detect the SNPs at the 3′-UTR of MTHFR gene. The SNPs with larger energy change were selected for preliminary experiments with randomly selected small samples (*n* = 30), and only the loci with polymorphisms in small ALL samples were further detected in all included samples.

### 2.3. DNA Extraction and Genotyping

Two milliliters of venous blood was obtained from each individual and then stored in tubes containing EDTA at −20°C. Genomic DNA was extracted using a Whole Blood Genomic DNA Extraction Kit (Takara, Dalian, China). DNA purity and concentrations were determined by spectrophotometric measurement of absorbance at 260 and 280 nm using a UV spectrophotometer. DNA samples were diluted into 10 mg/L and kept at −20°C before analysis.

The rs4846049 polymorphic site in MTHFR gene was genotyped by direct PCR sequencing. PCR was performed in 50 *μ*l reaction mixture, containing 1 *μ*l genomic DNA, 5 *μ*l 10 × PCR buffer, 1 *μ*l dNTPs (Fermentas, MD, USA), 5 *μ*l MgCL_2_, 1 *μ*l sense (5′-GTAGGTGTCAGCCCAATGC-3′), 1 *μ*l antisense (5′-GCGAGGAGAGTGGTTGTTG-3′) primers, 1 *μ*l Taq DNA polymerase (Fermentas, MD, USA), and 35 *μ*l pure H_2_O. PCR conditions were initial denaturation at 94°C for 3 min, followed by 40 cycles of denaturation at 94°C for 15 s, annealing at 55°C for 15 s, and extension at 72°C for 30 s, and a final extension at 72°C for 3 min. PCR products were purified using the MiniBEST DNA Fragment Purification Kit (Takara, Dalian, China) and sequenced on 3730XL DNA analyzer (Applied Biosystems, USA).

### 2.4. Quantitative Reverse Transcription-Polymerase Chain Reaction (qRT-PCR)

The qRT-PCR was performed to determine the expression level of MTHFR mRNA in blood samples of ALL patients and controls (>8 years) with the indicated genotypes (each, *n* = 2). Briefly, total RNA was isolated from blood using a TRIzol reagent (Thermo Fisher Scientific, Waltham, MA, USA) and reverse-transcribed into cDNA using the GoScript™ Reverse Transcriptase (Promega, Madison, WI, USA). PCR was run with 14 *μ*l reaction mixture, including 3 *μ*l cDNA, 7 *μ*l AceQ® qPCR SYBR® Green Master Mix (Vazyme Biotech Co., Piscataway, NJ, USA), 0.5 *μ*l sense (MTHFR: 5′-GTACAGCCGGACACACTGC-3′; *β*-actin: 5′-CCAACCGCGAGAAGATGA-3′), 0.5 *μ*l antisense (MTHFR: 5′-GCTGACTTCCAAGTCTCGTGT-3′; *β*-actin: 5′-CCAGAGGCGTACAGGGATAG-3′) primers, and 3 *μ*l pure H_2_O. qPCR amplification was carried out on the Bio-Rad CFX96 Real-Time PCR System C1000 Thermal Cycler (Bio-Rad, Hercules, CA) under conditions of 95°C for 4 min followed by 39 cycles of 95°C for 10 s, 60°C for 10 s, and 72°C for 20 s, and one cycle of 95°C for 10 s and 65°C for 5 s. Each sample was analyzed in triplicate. The relative expression of MTHFR was calculated using the 2^−ΔΔCt^ method.

### 2.5. Statistical Analysis

All statistical analyses were performed using the SPSS software (version 18.0; SPSS Inc., Chicago, IL, USA). Continuous variables were expressed using mean and standard deviation (SD), which were tested using the Student *t*-test, while categorical variables were displayed as frequency; the differences of which were performed using chi-squared test (Fisher's exact test). Odds ratios (OR) and corresponding 95% confidential intervals (95% CI) were calculated to estimate the relative risks. The association of all polymorphisms with ALL was studied using allele as well as codominant, dominant, recessive, and overdominant genotype models. *P* < 0.05 was considered to be significant.

## 3. Results

### 3.1. Patient Characteristics

The study recruited 110 pediatric patients diagnosed with ALL (72 males and 38 females, ages ranged from 9 months to 13 years) and 105 healthy controls (55 male and 50 female; ages ranged from 5 months to 15 years) between December 2017 and September 2018. The patients and control subjects were matched according to their gender (*P*=0.054) and age (*P* > 0.376), indicating they were comparable.

### 3.2. Rs4846049 Polymorphism Selection

**Figure 1:**
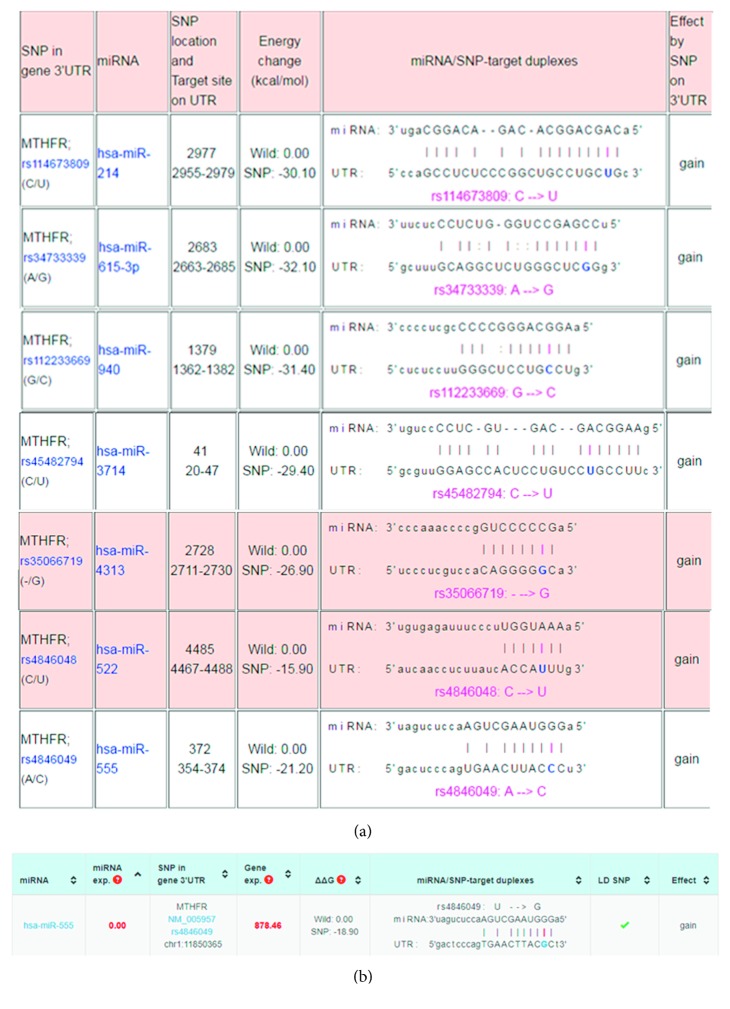
Selection of polymorphic loci of MTHFR that can be bound with miRNAs: (a) the prediction results on miRNASNP (http://www.bioguo.org/miRNASNP/) and (b) the prediction of rs4846049 on miRNASNP 2 (http://bioinfo.life.hust.edu.cn/miRNASNP2/).

**Figure 2:**
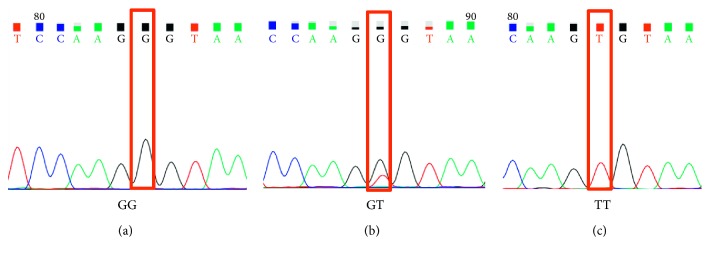
Chromatogram showing the rs4846049 polymorphism in the methylenetetrahydrofolate reductase (MTHFR) gene.

According to the prediction results by miRNASNP (Figure 1), the SNPs of rs114673809, rs34733339, rs112233669, rs4846048, rs4846049, rs35066719, and rs45482794 caused the binding energy between corresponding miRNA and MTHFR to be lower and the interaction of miRNA-mRNA may be more stable. In addition, the effects of these SNPs on MTHFR were to gain a miRNA binding site, and thus, the inhibition of MTHFR may be more significant. These SNPs were firstly detected in 30 samples to preliminarily confirm the polymorphisms of these SNPs in ALL patients. As a result, only rs4846049 at the binding site of miR-555 was found to have polymorphisms in ALL samples (Figure 2). Thus, only this SNP continued to be genotyped by direct PCR sequencing in the other 185 blood samples.

### 3.3. Associations between rs4846049 Polymorphism and ALL Risk

The allele and genotype frequencies of this studied polymorphism in ALL patients and controls are shown in Table 1. Overall, genotype distribution or allele carrier frequencies were not significantly different between patients with ALL and healthy controls (*P* > 0.05, Table 1). To further examine potential associations between rs4846049 and susceptibility to ALL, the patients were classified into two subgroups according to age and sex. The results showed that T allele may be a protective factor for ALL susceptibility (OR = 0.134, 95% CI = 0.028–0.639; *P*=0.005) in patients with age >8 years (Table 1). In line with the results of allele frequencies, the T allele (TT + GT) carriers were also observed to have a lower risk of ALL than the GG genotype carriers aged >8 years (OR = 0.133, 95% CI: 0.024–0.727; *P*=0.017) (Table 1). No associations in the allele and genotype frequencies were observed between the studied case and control groups in other subgroup analyses. Furthermore, we also specifically focused on the children with precursor B-cell ALL to further confirm the association of rs4846049. As expected, all the related findings were in line with the overall or subgroup analysis above, showing T allele was a protective factor for precursor B-cell ALL susceptibility (OR = 0.179, 95% CI = 0.037–0.863; *P*=0.022) in patients with age >8 years (Table 2).

### 3.4. Associations of rs4846049 Polymorphism and Clinical Features of ALL Patients

Table 3 summarizes the associations of rs4846049 polymorphism and clinical features of ALL patients. The results showed that the frequency of abnormal karyotypic change (details in supplementary information ([Supplementary-material supplementary-material-1])) was significantly higher in patients with the GG genotype (56.0%) compared to carriers of TT (0%) or GT (40.6%) genotypes, while C-myc break frequency was significantly higher in TT carriers (33%) than that in patients with GT (3.1%) or GG (0%) genotypes. No correlations of rs4846049 polymorphism were observed with other clinical characteristics.

### 3.5. Association of MTHFR Relative mRNA Expression and rs4846049 Polymorphism

**Figure 3:**
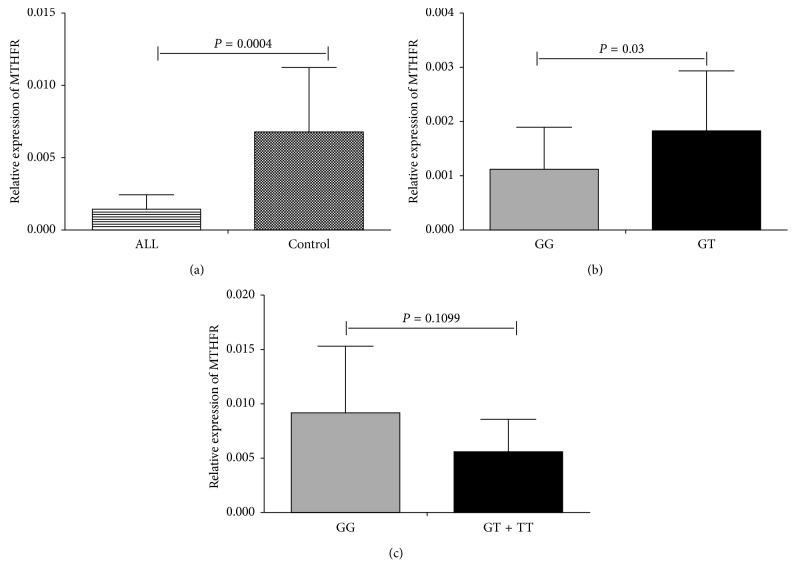
qRT-PCR for the assessment of the expression of MTHFR mRNA: (a) comparison between controls and ALL patients; (b) comparison between ALL patients with different genotypes of rs4846049; (c) comparison between controls with different genotypes of rs4846049. Data are presented as mean ± standard deviation. ALL, acute lymphoblastic leukemia; MTHFR, methylenetetrahydrofolate reductase. All data of acute lymphoblastic leukemia patients and controls are found in Supplementary information.

As shown in Figure 3(a), the relative mRNA expression of the MTHFR gene was significantly lower in ALL patients compared to healthy controls (*P*=0.0004), indicating polymorphisms that cause the low expression of MTHFR may be associated with ALL development. As expected, we found the ALL patients carrying the risk factor GG genotype exhibited the lower expression of MTHFR (*P*=0.03) (Figure 3(b)). The relative mRNA expression was not significantly different between GT and TT compared to GT genotype in controls (*P*=0.1099) (Figure 3(c)). These findings further explain the rs4846049 as the important mechanism for ALL development.

## 4. Discussion

The present study, for the first time, investigated the association between rs4846049 polymorphism of the MTHFR gene and the risk of ALL in Chinese children patients. The results showed the rs4846049 polymorphism (G allele or GG genotype) may increase the susceptibility to ALL in the population aged >8 years. miR-555 may more stably bind with GG genotype compared with GT and TT genotype and lead to the reduced expression of MTHFR in ALL.

Previously, there have studies to explore the association of MTHFR rs4846049 polymorphism and susceptibility to migraine [20], ischemic stroke [21], preeclampsia [22, 23], colorectal cancer [18], coronary artery disease [24, 25], attention-deficit/hyperactivity disorder [26], and cerebral palsy [27]. In the study of Salehi et al. [20], T allele (OR = 0.72, 95% CI = 0.56–0.93; *P*=0.01) and genotype of GT (OR = 0.61, 95% CI = 0.41–0.91; *P*=0.01), TT (OR = 0.57, 95% CI = 0.34–0.94; *P*=0.02), and GT + TT (OR = 0.60, 95% CI = 0.41–0.87; *P*=0.007) were suggested to be protective risk factors for migraine. Although no significant differences between preeclampsia and controls were observed, the ORs of TT and GT genotype were shown to be less than 1 and the expression of MTHFR was found to be relatively lower in patients with GG and GT genotype compared with TT [22], which indirectly explain the risk factor of G allele. Also, accumulating evidence has proved the MTHFR enzyme expression and activity were reduced and plasma homocysteine level was increased in patients with preeclampsia compared with normotensive pregnancy [28, 29]. Supplement of folic acid was demonstrated to resolve the attacks of migraine [30]. These were in accordance with the mechanisms of MTHFR in ALL [11–13], indirectly illustrating the creditability that rs4846049 polymorphism (G allele or GG genotype) led to reduced expression of MTHFR and promoted the development of ALL as reported in our study.

Several studies reported age at diagnosis of ALL was significantly correlated with clinical outcome, with the survival lower in infants (<1 year) and children aged ≥9 years compared with the intermediate age [31, 32]. Thus, timely identification of these patient group and schedule of effective treatment may be especially important. Moreover, a recent study indicated there was an association between IKZF1 rs4132601 polymorphism and age at diagnosis of childhood ALL, with the patients older in carriers of GT and TT genotypes in comparison with carriers of GG genotype [33]. Also, the GT and TT genotypes were found to be risky for the development of ALL in patients aged >9 years [34]. Thus, we also divided the patients into two groups according to the age range. In line with these studies, we also found G allele or GG + GT genotype that promoted the development of ALL may be a risk factor, particularly significant for patients with age >8 years (including 9-, 10-, 11-, 12-, and 13-year cases).

As described in the introduction, MTHFR plays important roles in methylation of DNA [6]. Its lower expression may lead to hypomethylation of centromeric and juxtacentromeric satellite DNA sequences and then induces karyotypic instability and chromosomal rearrangements, promoting the initiation of carcinogenesis [35, 36]. It is also reported that patients with complex karyotype alterations may have a poor prognosis [37, 38]. Thus, lower expressed MTHFR-induced karyotype abnormalities may be also a crucial mechanism for ALL. As expected, we found that the frequency of abnormal karyotypic change was significantly higher in the patients with the GG genotype that related to lower expression of MTHFR.

In addition, we also found c-myc break frequency was significantly higher in TT carriers (33%) than that of patients with GT (3.1%) or GG (0%) genotypes. C-myc has been widely recognized as a proto-oncogene [39]. Therefore, according to the above expression analysis on MTHFR, we hypothesized TT polymorphism may lead to the expression inhibition of c-myc and decrease the risk of ALL. This hypothesis seemed to be in line with the previous study about the positive relationship between MTHFR and c-myc (that is, knockdown of MTHFR (a risk factor for ALL) decreased lower levels of c-myc (protective role in ALL)) [40]. However, this result was not consistent with the known concept that DNA breaks can lead to translocations and gene amplification, fueling tumorigenesis [41]. We speculated this finding may be attributed to two reasons: (1) small sample size that may lead to the results unreliable; (2) c-myc gains defined as three or four copies of the gene that was previously demonstrated to have no effects on the prognosis in patients with lymphoma [42].

There were some limitations in this study. First, the sample size was relatively small, which may lead to the underestimation or overestimation of the associations. This was also a reason not to investigate the associations specifically in precursor T-cell ALL, a subtype of ALL with a poor prognosis. Second, there was a need to use the dual-luciferase assay to confirm the interaction between miR-555 and MTHFR. Third, the miRNASNP2 prediction analysis showed rs4846049 is a linkage disequilibrium SNP (Figure 1(b)), and thus, it is necessary to validate the combination effects of rs4846049 and other polymorphisms (such as TP53, rs8079544 [43]) on susceptibility to ALL. Fourth, further studies should be performed to analyze the association between the rs4846049 and response to treatment.

## 5. Conclusion

MTHFR rs4846049 polymorphism may be associated with increased risk of childhood ALL in the population aged >8 years and MTHFR mRNA expression. Further functional studies and large, well-designed clinical studies are still required to further elucidate the impact of rs4846049 polymorphisms on ALL.

## Figures and Tables

**Table 1:** Association of rs4846049 polymorphism and the risk of ALL.

		ALL (*n* = 110)	Control (*n* = 105)	*P* value	OR (95% CI)
Overall	Allele				
	G	182 (82.7)	165 (78.6)	—	1.000
	T	38 (17.3)	45 (21.4)	0.328	0.766 (0.474–1.238)
	Codominant				
	GG	75 (68.2)	67 (63.8)	—	1.000
	GT	32 (29.1)	31 (29.5)	0.880	0.922 (0.509–1.670)
	TT	3 (2.7)	7 (6.7)	0.201	0.383 (0.095–1.540)
	Dominant				
	GG	75 (68.2)	67 (63.8)	—	1.000
	GT + TT	35 (31.8)	38 (36.2)	0.565	0.823 (0.468–1.448)
	Recessive				
	GG + GT	107 (97.3)	98 (93.3)	—	1.000
	TT	3 (2.7)	7 (6.7)	0.207	0.393 (0.099–1.560)
	Overdominant				
	GG + TT	78 (70.9)	74 (70.5)	—	1.000
	GT	32 (29.1)	31 (29.5)	1.000	0.979 (0.544–1.762)

Age ≤8 years	Allele				
	G	150 (80.6)	135 (81.3)	—	1.000
	T	36 (19.4)	31 (18.7)	0.893	1.045 (0.613–1.782)
	Codominant				
	GG	60 (64.5)	56 (67.5)	—	1
	GT	30 (32.3)	23 (27.7)	0.619	1.217 (0.633–2.341)
	TT	3 (3.2)	4 (4.8)	0.713	0.700 (0.150–3.267)
	Dominant				
	GG	60 (64.5)	56 (67.5)	—	1
	GT + TT	33 (35.5)	27 (32.5)	0.751	1.141 (0.610–2.132)
	Recessive				
	GG + GT	90 (96.8)	79 (95.2)	—	1
	TT	3 (3.2)	4 (4.8)	0.708	0.658 (0.143–3.032)
	Overdominant				
	GG + TT	63 (67.7)	60 (72.3)	—	1
	GT	30 (32.3)	23 (27.7)	0.622	1.242 (0.650–2.375)

Age >8 years	Allele				
	G	32 (94.1)	30 (68.2)	—	1.000
	T	2 (5.9)	14 (31.8)	**0.005**	**0.134 (0.028–0.639)**
	Codominant				
	GG	15 (88.2)	11 (50.0)	—	1.000
	GT	2 (11.8)	8 (36.4)	0.065	0.183 (0.032–1.038)
	TT	0 (0.0)	3 (13.6)	0.100	1.273 (0.968–1.673)
	Dominant				
	GG	15 (88.2)	11 (50)	—	1.000
	GT + TT	2 (11.8)	11 (50)	**0.017**	**0.133 (0.024–0.727)**
	Recessive				
	GG + GT	17 (100.0)	19 (86.4)	—	1.000
	TT	0 (0.0)	3 (13.6)	0.243	1.158 (0.981–1.367)
	Overdominant				
	GG + TT	15 (88.2)	14 (63.6)	—	1.000
	GT	2 (11.8)	8 (36.4)	0.140	0.233 (0.042–1.293)

Male	Allele				
	G	117 (81.3)	88 (80.0)	—	1.000
	T	27 (18.7)	22 (20.0)	0.873	0.923 (0.493–1.728)
	Codominant				
	GG	47 (65.3)	36 (65.5)	—	1.000
	GT	23 (31.9)	16 (29.1)	0.846	1.101 (0.509–2.382)
	TT	2 (2.8)	3 (5.4)	0.652	0.511 (0.081–3.219)
	Dominant				
	GG	47 (65.3)	36 (65.5)	—	1.000
	GT + TT	25 (34.7)	19 (34.5)	1.000	1.008 (0.482–2.108)
	Recessive				
	GG + GT	70 (97.2)	52 (94.5)	—	1.000
	TT	2 (2.8)	3 (6.5)	0.652	0.495 (0.080–3.071)
	Overdominant				
	GG + TT	49 (68.1)	39 (70.9)	—	1.000
	GT	23 (31.9)	16 (29.1)	0.846	1.144 (0.533–2.457)

Female	Allele				
	G	65 (85.5)	77 (77.0)	—	1.000
	T	11 (14.5)	23 (230)	0.180	0.567 (0.257–1.249)
	Codominant				
	GG	28 (73.7)	31 (62.0)	—	1.000
	GT	9 (23.7)	15 (30.0)	0.471	0.664 (0.251–1.755)
	TT	1 (2.6)	4 (8.0)	0.366	0.277 (0.029–2.626)
	Dominant				
	GG	28 (73.7)	31 (62.0)	—	1.000
	GT + TT	10 (26.3)	19 (38.0)	0.264	0.583 (0.232–1.463)
	Recessive				
	GG + GT	37 (97.4)	46 (92.0)	—	1.000
	TT	1 (2.6)	4 (8.0)	0.384	0.311 (0.033–2.901)
	Overdominant				
	GG + TT	29 (76.3)	35 (70.0)	—	1.000
	GT	9 (23.4)	15 (30.0)	0.631	0.724 (0.277–1.895)

OR, odds ratios; CI, confidential intervals. Bold, significant results.

**Table 2:** Association of rs4846049 polymorphism and the risk of precursor B-ALL.

		ALL (*n* = 95)	Control (*n* = 105)	*P* value	OR (95% CI)
Overall	Allele				
	G	158 (83.2)	165 (78.6)	—	1.000
	T	32 (16.8)	45 (21.4)	0.256	0.743 (0.449–1.228)
	Codominant				
	GG	65 (68.4)	67 (63.8)	—	1.000
	GT	28 (29.5)	31 (29.5)	0.876	0.931 (0.504–1.721)
	TT	2 (2.1)	7 (6.7)	0.170	0.295 (0.059–1.471)
	Dominant				
	GG	65 (68.4)	67 (63.8)	—	1.000
	GT + TT	30 (31.6)	38 (36.2)	0.551	0.814 (0.452–1.465)
	Recessive				
	GG + GT	93 (97.9)	98 (93.3)	—	1.000
	TT	2 (2.1)	7 (6.7)	0.175	0.301 (0.061–1.487)
	Overdominant				
	GG + TT	67 (70.5)	74 (70.5)	—	1.000
	GT	28 (29.5)	31 (29.5)	1.000	0.998 (0.543–1.833)

Age ≤8 years	Allele				
	G	134 (81.7)	135 (81.3)	—	1.000
	T	30 (18.3)	31 (18.7)	1.000	0.975 (0.559–1.700)
	Codominant				
	GG	54 (65.9)	56 (67.5)	—	1
	GT	26 (31.7)	23 (27.7)	0.732	1.172 (0.597–2.301)
	TT	2 (2.4)	4 (4.8)	0.680	0.519 (0.091–2.948)
	Dominant				
	GG	54 (65.9)	56 (67.5)	—	1
	GT + TT	28 (34.1)	27 (32.5)	0.870	1.075 (0.563–2.055)
	Recessive				
	GG + GT	80 (97.6)	79 (95.2)	—	1
	TT	2 (2.4)	4 (4.8)	0.682	0.494 (0.088–2.773)
	Overdominant				
	GG + TT	56 (68.3)	60 (72.3)	—	1
	GT	26 (31.7)	23 (27.7)	0.612	1.211 (0.620–2.364)

Age >8 years	Allele				
	G	24 (92.3)	30 (68.2)	—	1.000
	T	2 (7.7)	14 (31.8)	**0.022**	**0.179 (0.037–0.863)**
	Codominant				
	GG	11 (84.6)	11 (50.0)	—	1.000
	GT	2 (15.4)	8 (36.4)	0.141	0.250 (0.043–1.454)
	TT	0 (0.0)	3 (13.6)	0.230	1.273 (0.968–1.673)
	Dominant				
	GG	11 (84.6)	11 (50)	—	1.000
	GT + TT	2 (15.4)	11 (50)	0.070	0.182 (0.032–1.018)
	Recessive				
	GG + GT	13 (100)	19 (86.4)	—	1.000
	TT	0 (0)	3 (13.6)	0.279	1.158 (0.981–1.367)
	Overdominant				
	GG + TT	11 (84.6)	14 (63.6)	—	1.000
	GT	2 (15.4)	8 (36.4)	0.259	0.318 (0.056–1.811)

Male	Allele				
	G	102 (81.0)	88 (80.0)	—	1.000
	T	24 (19.0)	22 (20.0)	0.871	0.941 (0.494–1.794)
	Codominant				
	GG	41 (65.1)	36 (65.5)	—	1.000
	GT	20 (31.7)	16 (29.1)	0.842	1.098 (0.495–2.431)
	TT	2 (3.2)	3 (5.4)	0.665	0.585 (0.093–3.702)
	Dominant				
	GG	41 (65.1)	36 (65.5)	—	1.000
	GT + TT	22 (34.9)	19 (34.5)	1.000	1.017 (0.476–2.173)
	Recessive				
	GG + GT	61 (96.8)	52 (94.5)	—	1.000
	TT	2 (3.2)	3 (6.5)	0.663	0.568 (0.091–3.532)
	Overdominant				
	GG + TT	43 (68.3)	39 (70.9)	—	1.000
	GT	20 (31.7)	16 (29.1)	0.842	1.134 (0.516–2.492)

Female	Allele				
	G	56 (87.5)	77 (77.0)	—	1.000
	T	8 (12.5)	23 (230)	0.106	0.478 (0.199–1.147)
	Codominant				
	GG	24 (75.0)	31 (62.0)	—	1.000
	GT	8 (25.0)	15 (30.0)	0.615	0.689 (0.251–1.892)
	TT	0 (0.0)	4 (8.0)	0.138	1.129 (1.002–1.272)
	Dominant				
	GG	24 (75.0)	31 (62.0)	—	1.000
	GT + TT	8 (25.0)	19 (38.0)	0.241	0.544 (0.203–1.453)
	Recessive				
	GG + GT	32 (100)	46 (92.0)	—	1.000
	TT	0 (0.0)	4 (8.0)	0.152	1.087 (1.002–1.180)
	Overdominant				
	GG + TT	24 (75.0)	35 (70.0)	—	1.000
	GT	8 (25.0)	15 (30.0)	0.802	0.778 (0.285–2.121)

OR, odds ratios; CI, confidential intervals. Bold, significant results.

**Table 3:** Association of rs4846049 polymorphism with clinical features of ALL patients.

	GG (*n* = 75)	GT (*n* = 32)	TT (*n* = 3)	*P* value
Male (*n*, %)	47 (62.7)	23 (71.9)	2 (66.7)	0.723
Age (mean)	5.60 ± 3.31	4.63 ± 2.89	6.33 ± 1.16	0.301
Patients with age >8 years (*n*, %)	15 (20.0)	2 (6.3)	0 (0.0)	0.163
WBC (×10^9^/L)	33.37 ± 78.92	28.40 ± 54.90	40.49 ± 65.31	0.929
Hb (g/L)	89.24 ± 19.68	87.91 ± 27.73	96.33 ± 29.01	0.818
Platelet (×10^9^/L)	115.35 ± 113.98	112.00 ± 107.67	113.00 ± 41.62	0.990
Organomegaly (*n*, %)	50 (66.7)	21 (65.6)	3 (100.0)	0.715
Immunophenotype				0.436
T (*n*, %)	9 (12.0)	3 (9.4)	1 (33.3)	0.455
B (*n*, %)	65 (86.7)	28 (87.5)	2 (66.7)	0.501
Mixture (*n*, %)	1 (1.3)	1 (3.1)	0 (0.0)	0.537
FISH detection				
C-myc break (*n*, %)	0 (0.00)	1 (3.1)	1 (33.3)	**0.017**
MLL break (*n*, %)	4 (5.3)	2 (6.3)	0 (0.0)	1.000
TCF3/PBX1fusion (*n*, %)	5 (6.7)	2 (6.3)	0 (0.0)	1.000
TEL-AML1fusion (*n*, %)	13 (17.3)	7 (2.2)	0 (0.0)	0.708
BCR, ABL1, ASS2 tricolour fusion (*n*, %)	3 (4.0)	0 (0.0)	0 (0.0)	0.589
Chromosome deficiency (*n*, %)	1 (1.3)	0 (0.0)	0 (0.0)	1.000
Normal (*n*, %)	48 (64.0)	20 (6.3)	2 (66.7)	1.000
Not done (*n*, %)	1 (1.3)	0 (0.0)	0 (0.0)	1.000
Karyotype changes				
Normal (*n*, %)	29 (38.7)	13 (40.6)	3 (100.0)	0.132
Abnormal (*n*, %)	42 (56.0)	13 (40.6)	0 (0.0)	0.023^*∗*^
Not done (*n*, %)	4 (5.3)	6 (18.8)	0 (0.0)	0.096
WHO classification				0.936
B-ALL, recurrent cytogenetic abnormalities (*n*, %)	26 (34.7)	14 (43.8)	2 (66.7)	0.336
B-ALL, *t* (9; 22) (q34.1; q11.2); BCR-ABL1 (*n*, %)	3 (4.0)	0 (0.0)	0 (0.0)	0.589
B-ALL, *t* (v;11q23.3); KMT2A rearrangement (*n*, %)	3 (4.0)	2 (6.2)	0 (0.0)	0.682
B-ALL, *t* (12; 21) (p13.2; q22.1); ETV6-RUNX1 (*n*, %)	13 (17.3)	6 (18.8)	0 (0.0)	1.000
B-ALL, high hyperdiploid (*n*, %)	16 (21.3)	5 (15.6)	0 (0.0)	0.884
B-ALL, *t* (1; 19) (q23; p13.3); TCF3-PBX1 (*n*, %)	6 (8.0)	2 (6.2)	0 (0.0)	1.000
T-ALL (*n*, %)	8 (10.7)	3 (9.4)	1 (33.33)	1.000
FAB classification				
L1 (*n*, %)	7 (9.3)	4 (12.5)	0 (0.0)	0.804
L2 (*n*, %)	51 (68.0)	21 (65.6)	2 (66.7)	0.921
L3 (*n*, %)	6 (8.0)	4 (12.5)	1 (33.3)	0.205
Unknown (*n*, %)	11 (14.7)	3 (9.4)	0 (0.0)	0.700
Lymphadenopathy (*n*, %)	55 (73.3)	22 (68.8)	2 (66.7)	0.843
CSF involvement (*n*, %)	0 (0.0)	1 (3.1)	0 (0.0)	0.318

WBC, white blood cell; Hb, hemoglobin; FAB, French-American-British; CSF, cerebrospinal fluid; FISH, fluorescence in situ hybridization. Bold, significant result at *P* < 0.05. ^*∗*^One-sided *P* value; the other were two-sided.

## Data Availability

All original data produced for the purpose of this project are available in supplementary information.

## Supplementary Materials

Supplementary Materials Raw data of patients and control: the clinical characteristics and SNP sequencing results of patients and control are included.
